# Baseline prevalence of high blood pressure and its predictors in a rural adult population of Bangladesh: Outcome from the application of WHO PEN interventions

**DOI:** 10.1111/jch.14386

**Published:** 2021-11-16

**Authors:** Lingkan Barua, Mithila Faruque, Shagoofa Rakhshanda, Palash Chandra Banik, Riffat Ara Shawon, Saidur Rahman Mashreky

**Affiliations:** ^1^ Department of Noncommunicable Diseases Bangladesh University of Health Sciences (BUHS) Dhaka Bangladesh; ^2^ Centre for Injury Prevention and Research Bangladesh (CIPRB) New DOHS Mohakhali Dhaka Bangladesh

**Keywords:** Bangladesh, hypertension, prehypertension, risk factors, WHO PEN

## Abstract

This cross‐sectional study estimated the prevalence of high blood pressure (BP) and examined its predictors at baseline following protocol 1 (actions 1 and 2) of World Health Organization (WHO) Package of Essential Noncommunicable Disease (PEN) Interventions in a selected rural area of Bangladesh. A total of 11 145 adults (both sex and age ≥ 18 years) completed both the questionnaire and clinical measurements at the household and community clinics, respectively. We defined high BP as systolic BP ≥ 120 mmHg or diastolic BP ≥ 80 mmHg, prehypertension (pre‐HTN) as systolic BP 120–139 mmHg or diastolic BP 80–89 mmHg, and hypertension (HTN) as systolic BP ≥ 140 mmHg or diastolic BP ≥ 90 mmHg and/or anti‐hypertensive drug intake for the raised BP. The prevalence of high BP was 51.2% (pre‐HTN, 25.3%; HTN, 25.9%). Among them, the proportion of pre‐HTN was higher among men (28.7%) while HTN was higher among women (27.4%). Other than fast food intake (pre‐HTN, OR: 1.110, *P *= .063) and women sex (HTN, OR: 1.236, *P *< .001), the pre‐HTN and HTN had higher odds for having same predictors as follows: age ≥ 40 years, family history of HTN, physical inactivity, central obesity, generalized obesity, and diabetes. In conclusion, the application of WHO PEN protocol 1 detected one‐fourth of the rural adult population had pre‐HTN and HTN respectively, and the common significant predictors of those were the age, family history of HTN, physical inactivity, generalized obesity, and diabetes.

## 1 INTRODUCTION

Raised blood pressure (BP) or hypertension is a major risk factor for noncommunicable diseases (NCD) like coronary artery disease (CAD), chronic kidney disease, and stroke.^1^ Among the NCD risk factors, raised BP attributed the highest proportion of global death compared to other NCD risk factors.^2^ Again, more than half (53%) of the cardiovascular disease (CVD) related mortality was contributed by hypertension. Thus, it is considered as the most prevalent NCD risk factor which is highly preventable.^3^, ^4^ Although HTN is mostly preventable, globally 22% of the adult population had raised BP in 2015 and 28% of this burden was distributed among less developed or low‐income countries.^5^ The prevalence of raised BP varied across the regions of the World Health Organization and South‐East Asia positioned (SEA) third place (25%) next to Africa (27%) and the Eastern Mediterranean region (26%).^6^ A systematic review and meta‐analysis conducted among the countries of the South Asian Association for Regional Cooperation (SAARC) reported that most of the studies of this region had inconsistent data on HTN and its risk factors.^7^ Hence, accurate and consistent estimation of HTN prevalence and its risk factors is crucial for prevention and management, especially in developing countries with low resources.

Bangladesh is currently in the third stage of epidemiological transition which is characterized by shifting from acute infectious and deficiency diseases to NCDs. The wave of this transition has also influenced the livelihood and health status of the rural population reported as an increase in chronic diseases.^8^ About 73% of the Bangladeshi population is living in rural areas where the population, healthcare infrastructure, and healthcare provider characteristics are very different compared to the urban setting.^9^ A study reported that most of the rural population had to depend on homeopathic physicians, local nurses, hospital compounders, the sellers of medical store, etc, during their medical emergencies.^10^ The same study also stated that strong beliefs on superstitions encouraged them to go to local Herbalists, Hakim or Kabiraj for a natural cure, who have limited knowledge on modern medical sciences.^10^ Regarding HTN, the evidence demonstrated that graduate doctors (MBBS and specialized) diagnosed only 53.5% of the HTN in rural areas of Bangladesh, and the remaining portion was diagnosed by unqualified healthcare providers. Again 26% of the diagnosed population discontinued their medication for HTN.^11^ These findings indicate that identification of HTN among the rural population of Bangladesh is inadequate and getting appropriate care and follow‐up is unlikely.

Previous studies reported that the prevalence of pre‐HTN and HTN among the rural Bangladeshi population ranged from 20.3% to 41.5%^12^, ^13^, ^14^, ^15^ and 6.9%‐30.6%,^12^, ^13^, ^14^, ^15^, ^16^ respectively. However, we have not found any study that evaluated the distribution of high BP based on the data collected following the WHO Package of Essential Noncommunicable Disease Interventions (WHO PEN).^17^ As WHO PEN is a globally acceptable cost‐effective protocol to address NCDs and its risk factors for prevention and management, several countries attempted to implement it. In Bangladesh, we first time attempted to apply the WHO PEN protocol 1 in primary care settings to gather the baseline data about major NCDs and their risk factors. Primarily only, the protocols for cardiovascular disease and diabetes were selected for implementation. To address the data gap, this study aimed to determine the distribution of high BP (pre‐HTN and HTN) and to examine the predictors of high BP (pre‐HTN and HTN) among the rural adult population in Bangladesh.

## 2 METHODS

### 2.1 Study design, setting, and population

This cross‐sectional study was conducted at baseline during the WHO PEN implementation in the Dhangara Union of Raiganj Upazila under the Sirajganj district, a rural area of the Rajshahi division (Figure S1). The Upazila comprises nine unions, which is the lowest administrative unit of a division. The Dhangara Union has 12 323 households comprising 51 759 populations, and among them, 35 704 were adult population (≥18 years old) during the data collection. We censused all the adult population of age 18 or above except those who were mentally challenged or institutionalized in a hospital, prison, nursing home, or other such institutions (supporting document 1). The study period was from January 2019 to June 2019.

### 2.2 Data collection procedure

A face‐to‐face interview was conducted at the household level using a mobile app developed based on a semi‐structured pre‐tested questionnaire. A group of well‐trained male and female data collectors (students of health sciences perusing BSc/MSc) interviewed the participants, measured their physical parameters, and assessed glycaemic status both at households and CCs respectively. The pre‐tested questionnaire was mainly prepared and adapted as per the STEP‐wise approach to Surveillance (STEPS) of NCD risk factors survey questionnaire of the World Health Organization (WHO, Version 3.2)^18^ and the “Action1 (ask about)” and “Action 2 (Assess)” sections of the WHO PEN protocol 1 (supporting document 2).^17^ We modified both the WHO STEPS and Action 1 of WHO PEN questionnaires to draft the final questionnaire used in the mobile app to collect information. The final drafted questionnaire has broadly two parts: one is asking questions and another is measurements. The “asking questions” part was prepared based on STEP 1 (demographic information and behavioral measurement) of the STEPS survey questionnaire and the “Action1 (ask about)” section of WHO PEN. At the household level, the “asking questions” part of the drafted questionnaire used to collect information about the participants’ sociodemographic status, existing comorbidities, family history of chronic diseases, behaviors (tobacco and alcohol consumption, physical activity, dietary habits, added salt intake), and metabolic risk factors (history of raised blood glucose, and raised blood pressure). Each participant was interviewed for approximately 25–30 minutes and then suggested to visit the nearby community clinic (CC) on the next day in the fasting state to assess physical and biochemical parameters.

The “measurements” part was prepared based on STEP 2 (physical measurements) and STEP 3 (biochemical measurements) of the STEPS survey questionnaire, and the “Action 2 (assess)” section of WHO PEN. In the CC, we used the “measurement” part of the drafted questionnaire to record anthropometric measurements, blood pressure, and capillary blood glucose levels. In this stage, standard weight scale (CAMRY, model BR2016, Hong Kong), height measurement scales, measuring tape, and automated digital BP machine (Omron, model JPN 2, Japan) were used to record the weight, height, waist circumference, and BP, respectively, of the respondents. The physical measurements were carried out according to the “Noncommunicable disease risk factors survey Bangladesh 2010.”^19^ To measure height, participants were requested to remove their footwear and headgear, if any. After that they were asked to stand with feet together, heels against the floor, knees straight, eyes were at the same level as the ear, and looking straight ahead facing the interviewer. Then, height was measured by using a height measuring plastic tape in centimeter (cm). To measure weight, participants had to stand still on a weighing scale putting on a firm and flat surface following removal of their footwear and light casual clothing. Then, weight was measured by the portable weighing scale in kilogram (kg). Waist circumference was measured by a plastic tape placing horizontally midway between the two points of the lowest rib margin and the top of the iliac crest.^19^ Fasting capillary glucose was measured by a standard glucometer (GlucoLeader Enhance, HMD BioMedical, Taiwan) with aseptic precautions. The BP was measured two times, first measurement was taken after 15 minutes of rest time, and the second measurement was taken three minutes after the first measurement. The mean of the two measurements was used to determine the final BP. All the physical measurements were carried out by the well‐trained male and female data collectors maintaining adequate privacy.

### 2.3 Definition of high BP and its risk factors

High BP is defined as systolic BP ≥ 120 mmHg or diastolic BP ≥ 80 mmHg.^20^ The pre‐HTN within this range was defined as systolic BP 120 mmHg to 139 mmHg or diastolic BP 80 mmHg to 89 mmHg.^19^ Again, the cut‐off for HTN was systolic BP ≥ 140 mmHg or diastolic BP ≥ 90 mmHg^20^ and/or documented anti‐hypertensive drug intake for the raised BP. Here, new cases were those diagnosed for the first time in the study, and old cases were those previously diagnosed by a health professional and/or documented anti‐hypertensive drug intake for the raised BP.

The current tobacco use, current alcohol consumption, inadequate fruit and/vegetable intake, low physical activity, and diabetes were categorized as per the definition used in the “Noncommunicable disease risk factors survey Bangladesh 2010.”^19^ Generalized obesity and central obesity were categorized as per the definition of WHO and the International Diabetic Federation respectively.^21^, ^22^ The added salt intake was defined as taking dietary salt during eating a meal.^23^ The fast food intake was defined as “food that can be prepared quickly and easily and is sold in restaurants and snack bars as a quick meal or to be taken out.”^24^, ^25^ The details of the definitions are added as a Supporting document 3.

### 2.4 Quality assurance

To maintain the quality control of the study, we applied several measures: (i) pre‐testing of the drafted questionnaire to detect any inconsistency, unclear wording, or unusually longer time taken to administer, and finalized after appropriate modification; (ii) pre‐survey training of the team members including investigators, supervisor, data collectors to outline the rationale of the study, and the procedures and potential difficulties associated with data collection; (iii) strict monitoring at the field level to closely monitor the data collection through field coordinators. Besides, for monitoring of the total system, a separate dashboard was created that was regularly monitored by the investigators (iv), the app was tested in the field for data synchronization and integration with the existing primary health care system, (v) use of show cards for a better understanding of the related risk factors of high BP, (vi) use of robust equipment for physical and biochemical measurements.

### 2.5 Ethical consideration

Data were collected after informed written consent was obtained. Ethical approval to conduct the study was taken from the Ethical Review Board of the Center for Injury Prevention and Research, Bangladesh (ERC number: CIPRB/ERC/2019/003) (Supporting document 4).

### 2.6 Statistical analysis

The data were entered in the pre‐designed Microsoft office excel format which was later imported into the statistical software Statistical Product and Service Solutions version 20.0 for Windows (SPSS, Inc., Chicago, IL, USA). Initially, we checked the data for incompleteness, inconsistency, missing data, coding errors, and any outliers. A total of 11 244 adults visited the community clinics and provided their measurements. However, based on the initial checking, 11 145 remained for analysis and reporting. To assess the distribution of high BP and its risk factors, categorical variables were presented using frequency and percentages, and the continuous variable was presented using mean and standard deviation. We also used error bars to represent the overall proportion of high BP, new and old cases of HTN. These descriptive analyses were applied to achieve the first objective of this study (distribution of high BP) according to age, sex, and risk factors.

To identify the predictors of pre‐HTN and HTN, we used multinomial logistic regression analysis. For this, first, we checked the assumptions of regression analysis that included multicollinearity, outlier, normality, linearity, homoscedasticity, and the independence of observations. We did not find any violation of these assumptions. To identify the variables eligible to be included in the multinomial logistic regression analysis, univariate analyses were computed and a *P*‐value of ≤.25 was considered as the cut‐off. We calculated odds ratios (OR) and the factors that had OR > 1 was presented in the regression table for each outcome variable. This inferential statistic was applied to achieve the second objective of this study (predictors of high BP). All the estimates of precision were presented at a 95% confidence interval (CI), as appropriate. The statistical tests were considered significant (two‐sided) at a level of *P* < .05.

## 3 RESULTS

### 3.1 Background information of the study population

Table 1 depicted the background sociodemographic information of the study population. Here, the mean (SD) age of the study population was 44.8 (15.8) years and the age group of 41–50 years was the highest proportion (22.4%) among other age categories. The majority of the participants were women (66.4%), married (83.8%), and housewives (53.6%), and completed secondary school (49.4%).

**TABLE 1:** Sociodemographic status of the study population, No. = 11 145

**Information**	**No. (%)**	**95% CI**
**Age (years), mean ± SD**	44.8 ± 15.8	
**Age categories (years)**		
≤ 30	2204 (19.8)	19.1 – 20.5
31 ‐ 40	2437 (21.9)	21.1 – 22.7
41 ‐ 50	2501 (22.4)	21.6 – 23.2
51 ‐ 60	2105 (18.9)	18.2 – 19.6
> 60	1898 (17)	16.3 – 17.7
**Gender**		
Men	3749 (33.6)	32.7 – 34.5
Women	7396 (66.4)	65.5 – 67.3
**Educational status**		
Illiterate	3995 (35.8)	34.9 – 36.7
Primary	1288 (11.6)	11 – 12.2
Secondary	5510 (49.4)	48.5 – 50.3
Higher education	352 (3.2)	2.9 – 3.5
**Marital status**		
Unmarried	780 (7)	6.5 – 7.5
Married	9339 (83.8)	83.1 – 84.5
Widow	930 (8.3)	7.8 – 8.8
Others	96 (0.9)	0.7 – 1.1
**Occupational status**		
Unemployed	943 (8.5)	8 ‐ 9
Service holder	311 (2.8)	2.5 – 3.1
Farmer	1362 (12.2)	11.6 – 12.8
Businessmen	694 (6.2)	5.8 – 6.6
Self‐employed	1736 (15.6)	14.9 – 16.3
Housewife	5971 (53.6)	52.7 – 54.5

SD, standard deviation; CI, confidence interval.

### 3.2 Age‐ and sex‐specific distribution of high BP among the study population

**FIGURE 1:**
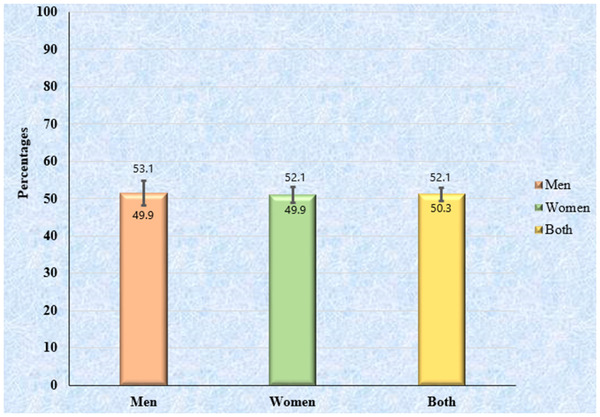
Prevalence of high blood pressure (BP) among the rural adult population of Bangladesh (N = 11 145)

**FIGURE 2:**
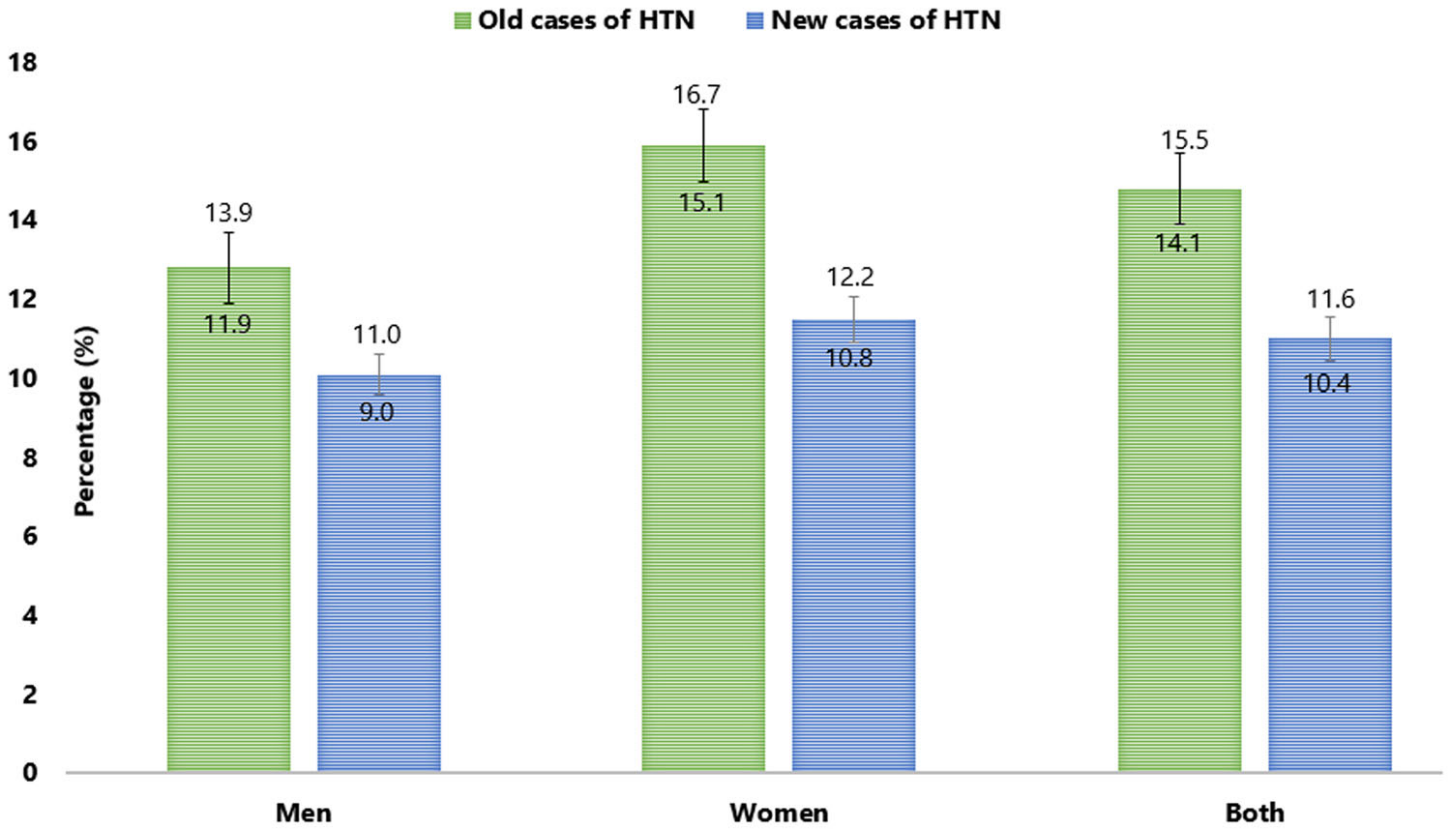
Proportion of old and new cases of hypertension among the rural adult population of Bangladesh (N = 11 145)

Overall, 51.2% of participants had high BP without any sex differences (Figure 1). Among the participants with high BP, the overall proportion of pre‐HTN (25.3%) and HTN (25.9%) were similar (Table 2). The sex‐wise distribution showed that the proportion of individuals with pre‐HTN was higher among men while HTN was higher among women (Table 2). Again, among the hypertensive population, the proportion of new and old cases were 14% and 11.8%, respectively. However, the proportion of old cases of HTN was more prevalent among women (15.9%) compared to men (12.8%) (Figure 2). According to age‐wise distribution, the proportion of individuals with pre‐HTN was higher than the HTN below the age of 50 years and after the age of 50 years, this relationship reversed. The proportion of people with HTN gradually increased with advancing age and became maximum (48.9%) after the age of 60 years (Table 2).

**TABLE 2:** Age‐and sex‐wise distribution of blood pressure among Bangladeshi rural adult population, no. = 11 145

		**Normal blood pressure** *		**Pre‐hypertension** **^†^**		**Hypertension** **^‡^**	
**Age groups (years)**	**No**.	**%**	**95% CI**	**%**	**95% CI**	**%**	**95% CI**
**Men**							
≤ 30	678	61.7	58.0 – 65.4	34.5	30.9 – 38.1	3.8	2.4 – 5.2
31 – 40	719	57.2	53.4 – 60.8	30.6	27.2 ‐ 34	12.2	9.7 – 14.5
41 – 50	794	51.8	48.3 – 55.3	28.2	25.1 – 31.3	20.0	17.2 – 22.8
51 – 60	797	39.5	36.1 – 42.9	30.1	26.9 – 33.3	30.4	27.2 – 33.6
> 60	761	34.4	31.0 – 37.8	20.8	17.9 – 23.7	44.8	41.3 – 48.3
Total	3749	48.5	46.9 – 50.1	28.7	27.3 – 30.1	22.8	21.5 – 24.1
**Women**							
≤ 30	1526	73.5	71.3 – 75.7	20.7	18.7 – 22.7	5.8	4.6 ‐ 7
31 – 40	1718	58.8	56.5 – 61.1	25.5	23.4 – 27.6	15.7	14 – 17.4
41 – 50	1707	43.5	41.2 – 45.9	26.7	24.6 – 28.8	29.8	27.6 ‐ 32
51 – 60	1308	33.6	31.0 – 36.2	21.5	19.6 – 23.4	44.9	42.2 – 47.6
> 60	1137	27.2	24.6 – 29.8	22.4	20 – 24.8	50.4	47.5 – 53.3
Total	7396	49.0	47.9 – 50.1	23.6	22.6 – 24.6	27.4	26.4 – 28.4
**Both genders**							
≤ 30	2204	69.9	68.0 – 71.8	25.0	23.2 – 26.8	5.2	4.3 – 6.1
31 – 40	2437	58.4	56.4 – 60.4	27.0	25.2 – 28.8	14.6	13.2 ‐ 16
41 – 50	2501	46.1	44.1 – 48.1	27.2	25.5 – 28.9	26.7	25 – 28.4
51 – 60	2105	35.9	33.9 – 37.9	24.8	23 – 26.6	39.4	37.3 – 41.5
> 60	1898	30.1	28.0 – 32.2	21.8	19.9 – 23.7	48.2	46 – 50.4
Total	11145	48.8	47.9 – 49.7	25.3	24.5 – 26.1	25.9	25.1 – 26.7

* Systolic blood pressure < 120 mmHg and diastolic blood pressure < 80 mmHg.

^†^ Systolic blood pressure 120 – 139 mmHg or diastolic blood pressure 80 ‐89 mmHg.

^‡^ Systolic blood pressure ≥ 140 mmHg and/ diastolic blood pressure ≥ 90 mmHg.

### 3.3 Risk factor‐specific distribution of high BP among the study population

Inadequate fruit/vegetable intake (68.5%) and added salt intake (62.8%) were the highly prevalent risk factors of high BP among the study population (Table 3).

**TABLE 3:** Overall prevalence of high BP risk factors and distribution of BP for each risk factors among the study population, no. = 11 145

**Risk factors**	**Total**	**Men**						**Women**					
		**Normal BP** *		**Pre‐HTN** **^†^**		**HTN** **^‡^**		**Normal BP** *		**Pre‐HTN** **^†^**		**HTN** **^‡^**	
	**%**	**%**	**95% CI**	**%**	**95% CI**	**%**	**95% CI**	**%**	**95% CI**	**%**	**95% CI**	**%**	**95% CI**
Tobacco use	35.6	51.7	49.4 ‐ 54	26.7	24.7 – 28.7	21.6	19.7 – 23.5	43.4	41.3 – 45.5	22.3	20.5 – 24.1	34.3	32.3 – 36.3
Intake of unhealthy diets													
Inadequate fruit/vegetable	68.5	50.1	48.1 – 52.1	26.7	24.9 – 28.5	23.2	21.5 – 24.9	49.0	47.7 – 50.3	23.5	22.4 – 24.6	27.5	26.3 – 28.7
Fast food	31.3	51.3	48.8 – 53.8	31.9	29.6 – 34.2	16.8	14.9 – 18.7	53.7	51.5 – 55.9	25.3	23.4 – 27.2	21.0	19.2 – 22.8
Sweetened beverage	36.6	49.7	47.5 – 51.9	29.7	27.7 – 31.7	20.7	18.9 – 22.5	54.9	52.7 – 57.1	24.5	22.6 – 26.4	20.7	18.7 – 22.1
Added salt	62.8	52.8	50.7 – 54.9	29.3	27.4 – 31.2	17.9	16.3 – 19.5	51.4	50.0 – 52.8	24.6	23.4 – 25.8	24.0	22.8 – 25.2
Alcohol intake (once in life)	0.6	60.4	46.6 – 74.2	29.2	16.3 – 42.1	10.4	1.8 ‐ 19	50.0	29.1 – 70.9	31.8	12.3 – 51.3	18.2	2.1 – 34.3
Physically inactive	18.1	39.2	35.8 – 42.6	30.2	27.0 – 33.4	30.7	27.5 – 33.9	41.7	38.9 – 44.4	23.3	20.9 – 25.7	35.0	32.3 – 37.7
Generalized obesity	3.5	20.0	8.9 – 31.1	32.0	19.1 – 44.9	48.0	34.2 – 61.8	23.5	19.0 – 28.0	30.8	25.9 – 35.7	45.7	40.4 ‐ 51
Diabetes	9.2	25.3	21 – 29.6	24.5	20.2 – 28.8	50.1	45.2 ‐ 55	20.1	17.7 – 24.1	23.5	20.2 – 26.8	55.6	51.7 – 59.5
Familial HTN	26.5	44.3	41.1 – 47.5	30.9	28 – 33.8	24.8	22.1 – 27.5	45.0	42.8 – 47.2	24. 8	22.9 – 26.7	30.1	28.1 – 32.1

BP, blood pressure; HTN, hypertension.

* Systolic blood pressure < 120 mmHg and diastolic blood pressure < 80 mmHg.

^†^ Systolic blood pressure 120 – 139 mmHg or diastolic blood pressure 80 ‐89 mmHg.

^‡^ Systolic blood pressure ≥ 140 mmHg and/ diastolic blood pressure ≥ 90 mmHg.

Here (Table 3), the risk factors‐wise distribution showed sex‐specific differences for pre‐HTN, but not for the HTN. In men, pre‐HTN was higher among those with generalized obesity (32%), consumed fast food (31.9%), did less physical activity (30.2%), and had a family history of HTN (30.9%). In women, pre‐HTN was higher among the participants who had a history of alcohol intake at least once in their lifetime (31.8%) and generalized obesity (30.8%). However, in both sexes, the prevalence of hypertension was higher among the participants with diabetes (men, 50.1%; women, 55.6%) and generalized obesity (men, 48%; women, 45.7%) (Table 3).

### 3.4 Predictors of high BP among the study population

The univariate analysis shows highly significant (*P *< .001) association for most of the risk factors of high BP (Table S1). Based on the univariate analysis, multinomial logistic regression was applied in Table 4 that shows the predictors of high BP (pre‐HTN and HTN) among the study population. Other than fast food intake (pre‐HTN, OR: 1.110, CI: 0.994‐1.239, *P *= .063) and women sex (HTN, OR: 1.236, CI: 1.099‐1.391, *P *< .001), the pre‐HTN and HTN had higher odds for having same risk factors as follows: age ≥ 40 years, family history of HTN, physical inactivity, central obesity, generalized obesity, and diabetes.

**TABLE 4:** Predictors of high blood pressure (pre‐hypertension and hypertension) among Bangladeshi rural adults, no. = 11 145

**High BP**	**Factors**		**B**	***P*‐value** *	**OR**	**95% CI for OR**	
						**Lower Bound**	**Upper Bound**
Pre‐hypertension^†^	Age						
	≥ 40 years		0.528	< .001	1.696	1.522	1.889
	< 40 years	*Ref*.					
	Family history of HTN						
	Present		0.259	< .001	1.295	1.163	1.442
	Absent	*Ref*.					
	Fast food intake						
	Yes		0.104	.063	1.110	0.994	1.239
	No	*Ref*.					
	Physical activity						
	Inactive		0.261	<.001	1.298	1.147	1.468
	Active	*Ref*.					
	Central obesity						
	Present		0.049	.353	1.050	0.947	1.164
	Absent	*Ref*.					
	Generalized obesity						
	Present		1.040	<.001	2.829	2.136	3.749
	Absent	*Ref*.					
	Diabetes						
	Present		0.600	<.001	1.823	1.507	2.204
	Absent	*Ref*.					
Hypertension^‡^	Age						
	≥ 40 years		1.922	< .001	6.833	5.988	7.796
	< 40 years	*Ref*.					
	Sex						
	Women		0.212	< .001	1.236	1.099	1.391
	Men	*Ref*.					
	Family history of HTN						
	Present		0.484	< .001	1.622	1.444	1.822
	Absent	*Ref*.					
	Physical activity						
	Inactive		0.521	<.001	1.683	1.483	1.910
	Active	*Ref*.					
	Central obesity						
	Present		0.088	.125	1.092	0.976	1.221
	Absent	*Ref*.					
	Generalized obesity						
	Present		1.389	<.001	4.011	3.018	5.332
	Absent	*Ref*.					
	Diabetes						
	Present		1.235	<.001	3.437	2.891	4.086
	Absent	*Ref*.					

OR, odd ratio; CI, confidence interval; BP, blood pressure; HTN, hypertension.

* Significant at the threshold of *P* < 0.05.

^†^ Systolic blood pressure 120 – 139 mmHg or diastolic blood pressure 80 ‐89 mmHg.

^‡^ Systolic blood pressure ≥ 140 mmHg and/ diastolic blood pressure ≥ 90 mmHg .

## 4 DISCUSSION

In Bangladesh, several studies have widely evaluated high BP in terms of pre‐HTN and HTN. However, those evaluations did not follow any recommended guidelines for screening, prevention, and management of populations with high BP. Again, most of those published studies did not target the rural population and utilized the root level primary healthcare facilities namely “community clinic (CC)” as the center of data collection. According to the health care delivery system of Bangladesh, these CCs may serve as the first health facility from where the individual with chronic diseases will be referred to the higher centers for subsequent treatment as per WHO PEN protocol. This is the first study of Bangladesh that presented pre‐HTN, HTN, and its predictors from the baseline data of globally recommended WHO PEN interventions that applied in the rural CCs where health services are maintaining with limited resources. We found that more than half of the study population residing in a rural area of Bangladesh had high BP and among them, one‐fourth (25.3%, 25.9%) had pre‐HTN and HTN respectively.

In Bangladesh, previous studies reported the prevalence of pre‐HTN as 31.9%^14^ and 41.5%^13^ among the rural adults, which were higher than the current study (25.3%). Again, in this study, the prevalence of pre‐HTN was higher among men than the women which are similar to other studies of Bangladesh.^13^, ^14^ Our study shows that the prevalence of pre‐HTN declined after the age of 50 years, which is also supported by an aforementioned study conducted in the rural setting.^14^ Both in the public health and clinical field, pre‐HTN carry great importance as interventions at this stage can prevent advancement to sustained HTN in the future and subsequent complications. Published literature reported that people with pre‐HTN were more likely to have masked HTN than people with optimal blood pressure. As a result, people with pre‐HTN are more likely to develop target end‐organ damage even before developing sustained HTN.^26^ Most recent evidence using the 2017 American College of Cardiology/American Heart Association BP guideline also showed that the CVD incidence rates were higher among those with elevated BP (41.5 per 100 000 person‐years) (pre‐HTN as per JNC 7 guideline) than those with normal BP (28.6 per 100 000 person‐years).^27^ However, pre‐HTN is not globally considered as a diagnostic category as most of these healthy individuals could be stigmatized as sick and treated with unnecessary medications.^28^ Nonetheless this criticism, pre‐HTN is a convergence point for several cardiovascular risk factors and an early can be valuable to track and sensitize people to adopt a healthy lifestyle.^29^ Thus, it is important to control BP among the patients with pre‐HTN and recognize it as a public health problem by the policymakers to mitigate the burden of HTN.

In a rural context, the current study detected more participants as hypertensive (25.9%) than the most recent national STEPS survey 2018 of Bangladesh (19.8%).^29^ Our finding is also higher than the previous STEPS survey 2010 of Bangladesh (17.9%),^19^ and a rural community‐based study (16%) that followed WHO STEPS survey protocol.^14^ The baseline prevalence of HTN varied in the countries that implemented the WHO PEN interventions. Among them, a higher baseline prevalence was reported by Gaza (64.3%) and Myanmar (35.2%),^30^, ^31^ and lower prevalence was reported by Bhutan (22.6%) and Democratic People's Republic of Korea (14.8%).^32^, ^33^ The proportion of HTN detected in this study is higher than the recent global data (17.5%),^34^ and lower than the previous data (31.1%) of the year 2000–2010.^35^ In our study, the proportion of HTN was higher among women which is also consistent with other rural studies of Bangladesh^13^, ^14^ and evidence from an Iranian study.^36^ Regarding detection of old and new cases, STEPS survey Bangladesh 2010 detected more old cases (18.6%), and STEPS survey Bangladesh 2018 detected a similar proportion (13.7%) of old cases of HTN as the current study.^19^, ^29^

In this study, unhealthy dietary habit (inadequate fruit/vegetable intake and added salt intake) was detected as the highly prevalent risk factors of high BP. In this regard, a recent review also reported the unhealthy diet and high sodium intake as the main culprit of sustain high BP.^35^ The distribution of pre‐HTN risk factors shows notable differences between sexes. In men, the prevalence of pre‐HTN was higher among the participants who had generalized obesity, consumed fast food, a family history of HTN, and inadequate physical activity. Previous studies also reported a high prevalence of pre‐HTN among the participants with generalized obesity,^37^, ^38^ family history of HTN,^39^, ^40^ and physical inactivity.^41^ We have not found any study that reported the burden of pre‐HTN concerning fast food consumption to compare our findings. In women, pre‐HTN was higher among those with a history of alcohol intake and who were generally obese. These findings are also consistent with a study conducted among Tanzanian rural women for alcohol intake and generalized obesity.^42^Although pre‐HTN shows the sex‐specific difference in its distribution, HTN shows a high prevalence among the participants of both genders with diabetes and generalized obesity. This finding is consistent with a rural study of Bangladesh.^15^

The current study identifies some non‐modifiable risk factors as the predictors of pre‐HTN and HTN. These include age and family history of HTN for both pre‐HTN and HTN and sex for HTN only. Previous community‐based rural studies of Bangladesh also determined age as a predictor of both pre‐HTN and HTN.^14^, ^15^ Other Asian countries also reported similar findings of age and high BP in terms of pre‐HTN and HTN.^36^, ^43^ A previous Bangladeshi study showed a significant positive relationship between high BP and age, irrespective of gender.^13^ Understanding the impact of age‐related BP increase in a population is of obvious clinical importance. This is also important for a country that is undergoing a rapid epidemiological transition in the rural areas and their life expectancy is increasing with time. However, it is not clear whether the age‐related BP increase is due to adaptation of modern lifestyle or these changes impact everyone equally or just high‐risk subpopulations.^44^ Hence, it demands further studies in a similar setting. Other than age, our finding of family history of HTN and high BP is also supported by other studies for pre‐HTN^36^ or HTN.^36^, ^43^ An original study that attempted to investigate the influence of the parental history of HTN on BP elucidated that the history of HTN in at least one parent was associated with offspring's HTN.^45^ These findings suggest that parental history of HTN should not be overlooked during medical examinations so that early detection and treatment of hypertension might be possible in current practice. We found sex (women) as another non‐modifiable predictor of HTN and it is supported by a national study of Bangladesh.^46^ A systematic review and meta‐analysis about the prevalence of HTN in Bangladesh also reported a similar finding as current study.^47^ Although globally, the prevalence of HTN is greater among men than women, in a rural setting like the current study, the sex‐specific difference in distribution of pre‐HTN and HTN are different maybe because of both biological (family milieu/body composition) and behavioral factors. In our study, family history of HTN (27% in women versus 25% in men), added salt intake (65.6% in women versus 57.3% in men), and obesity (4.6% in women versus 1.3% in men) are higher among women than men that may be contributing to the higher burden of HTN in women. Again, enrollment of high proportion of women than men (66.4% versus 33.6%) and illiteracy (38.5% in women versus 30.6% in men) are also responsible for such a higher burden of HTN in women. The higher prevalence of pre‐HTN in men than women may be because of fast food consumption (41.6% in men versus 26.1% in women), less physical activity (21.6% in men versus 16.4% in women), and diabetes (10.2% in men versus 8.8% in women).

Among the behavioral risk factors, fast food intake showed higher odds of pre‐HTN compared to having normal BP. Globally, data of association between fast food consumption and high BP in the adult is scanty. In this regard, an epidemiological study of Bangladesh reported a high percentage of fast food consumption (82%) as the risk factors of obesity and HTN among university students.^48^ However, we have not found any straightforward evidence that reported a significant association between fast food consumption and high BP. Interestingly, in children, no association was found between fast food intake and high BP which contradicts our study.^49^

Inadequate physical activity is another behavioral risk factor that showed higher odds of pre‐HTN compared to having normal BP. A prior study in a rural setting of Bangladesh also found a significant association between physical inactivity and HTN prevalence.^16^ Another Brazilian study also reported a higher prevalence of HTN among the participants who were physically inactive than those who were active.^50^ Generally, the proportion of developing high blood pressure is 30–50% higher among those who are physically inactive.^51^ Physical inactivity increase weight gain, which in turn increases the risk of high BP.^52^

Two modifiable metabolic risk factors namely obesity (generalized/central) and diabetes are found to be significant predictors of pre‐HTN and HTN in the current study. Previous studies in Bangladesh also reported similar findings for pre‐HTN and HTN in rural settings about obesity.^14^, ^15^, ^16^ However, in previous research, the association of central obesity (central fat distribution) measured using waist‐to‐hip ratio showed a stronger relationship with high BP than generalized obesity.^53^ Regarding diabetes, our findings coincide with another rural study in Bangladesh.^15^ The coexistence of diabetes and high BP is fatal as it causes additive increases in the risk of life‐threatening cardiovascular events.^54^

Overall, in a rural setting, using standard tools and rigorous protocol (WHO PEN protocol 1), we found a considerably high prevalence of high BP (pre‐HTN/HTN) and associated risk factors. It could be explained in the light of the epidemiological transition occurring in the rural areas of Bangladesh. An important study regarding epidemiological transition in rural Bangladesh reported a huge transition of increased mortality (3527% for cardiovascular diseases and cerebrovascular diseases, and 495% for malignant neoplasms) from infectious diseases to noncommunicable diseases.^8^ This massive increase in mortality particularly due to CVD suggesting that some changes may be happening in diet and lifestyle among the rural population of Bangladesh that needs urgent attention.

Our study has several strengths. For the first time in Bangladesh, high BP and its risk factors are reported using the baseline data of globally recommended WHO PEN protocol1 and using root level health centers like CCs. We assume, the action‐oriented protocol 1 of WHO PEN is more convenient and provided a valid estimate of high BP in resource constrain rural settings like Bangladesh. The inclusion of some emerging risk factors like fast food consumption, family history, etc, in the evaluation of high BP in the Bangladesh context carries great importance since prior evidence is scanty.

## 5 LIMITATIONS

The study findings should be interpreted in light of some limitations. First, our study is a cross‐sectional study in design; it only provides association and not causation. Second, the participating CCs were not representative of all CCs of Bangladesh, considering their geographical distribution, as some were underrepresented or not represented at all. Therefore, the results of our study cannot be extrapolated to the all rural population in Bangladesh. Again, this study followed the recommended referral system of WHO PEN protocol where participants were referred to the CC from the household level. However, the current findings of high BP are difficult to be compared with a non‐intervention area where many individuals bypass the CCs and directly visit the upper level of health facilities for screening and treatment. Third, self‐reported statements of behavioral risk factors may be subjected to recall bias. Finally, as the data is skewed towards women, it may underestimate the actual prevalence of high BP among the study population.

## 6 CONCLUSION

Half of the selected rural adult population of Bangladesh has high blood pressure. Again, one‐fourth of them have pre‐HTN and HTN. The equal burden of pre‐HTN and HTN in a rural setting is alarming as it will increase the total healthcare expenditure (direct/indirect) in near future among the rural deprived population. Again, as their health‐seeking behavior is poor, they rarely visit the nearby health facilities to identify themselves as at risk of high BP and subsequent complications. Moreover, as an economically poor community, they have limited access to get the advanced health services to mitigate the risk and manage the related complications. All of these demands a cost‐effective protocol like WHO PEN to include these high‐risk populations under a unique screening program. Here, the identified people with pre‐HTN are the target of future intervention to reduce the subsequent HTN and its complications. Besides, we recommend implementing the WHO PEN protocol 1 in other regions of the country and various settings to generate comparable data and assess its effectiveness that will help to adopt the new protocol within the existing health care system.

## CONFLICT OF INTEREST

Authors have no conflicts of interest to disclose.

## AUTHOR CONTRIBUTIONS

Conception, LB, RAS, SR, MF, PCB, and SRM; design of the work, LB, PCB, MF, and SRM; acquisition of data, LB, RAS, SR, and PCB; Analysis, LB and PCB; interpretation, LB, PCB, MF, and SRM; drafting; LB, critical revision, RAS, SR, PCB, MF, and SRM. All authors have read and approved the final version.

## Supporting information

Supporting information
**Supportive document 1**: Flow Chart of study methods applied to collect data from selected rural population of Bangladesh

Supporting information
**Supportive document 2**: WHO PEN Intervention

Supporting information
**Supportive document 3**: Implementation of WHO PEN intervention phases

Supporting information
**Supportive document 4**: Ethical approval letter

Supporting information
**Supportive Figure S1**: Map of Bangladesh & the study site

Supporting information
**Supportive Table S1**: Univariate analysis (χ^2^‐test) between the blood pressure categories (normal/pre‐hypertension/hypertension) and the risk factors among Bangladeshi rural adults, n = 11145
