# Supplementary material for: Baseline prevalence of high blood pressure and its predictors in a rural adult population of Bangladesh: Outcome from the application of WHO PEN interventions
Source: J Clin Hypertens (Greenwich). 2021 Nov 16;23(12):2042–52. doi: 10.1111/jch.14386 (PMC8696237; doi:10.1111/jch.14386)
Supplement: Supplementary file 1 — Supporting information Supportive document 1: Flow Chart of study methods applied to collect data from selected rural population of Bangladesh [file JCH-23-2042-s003.pdf]

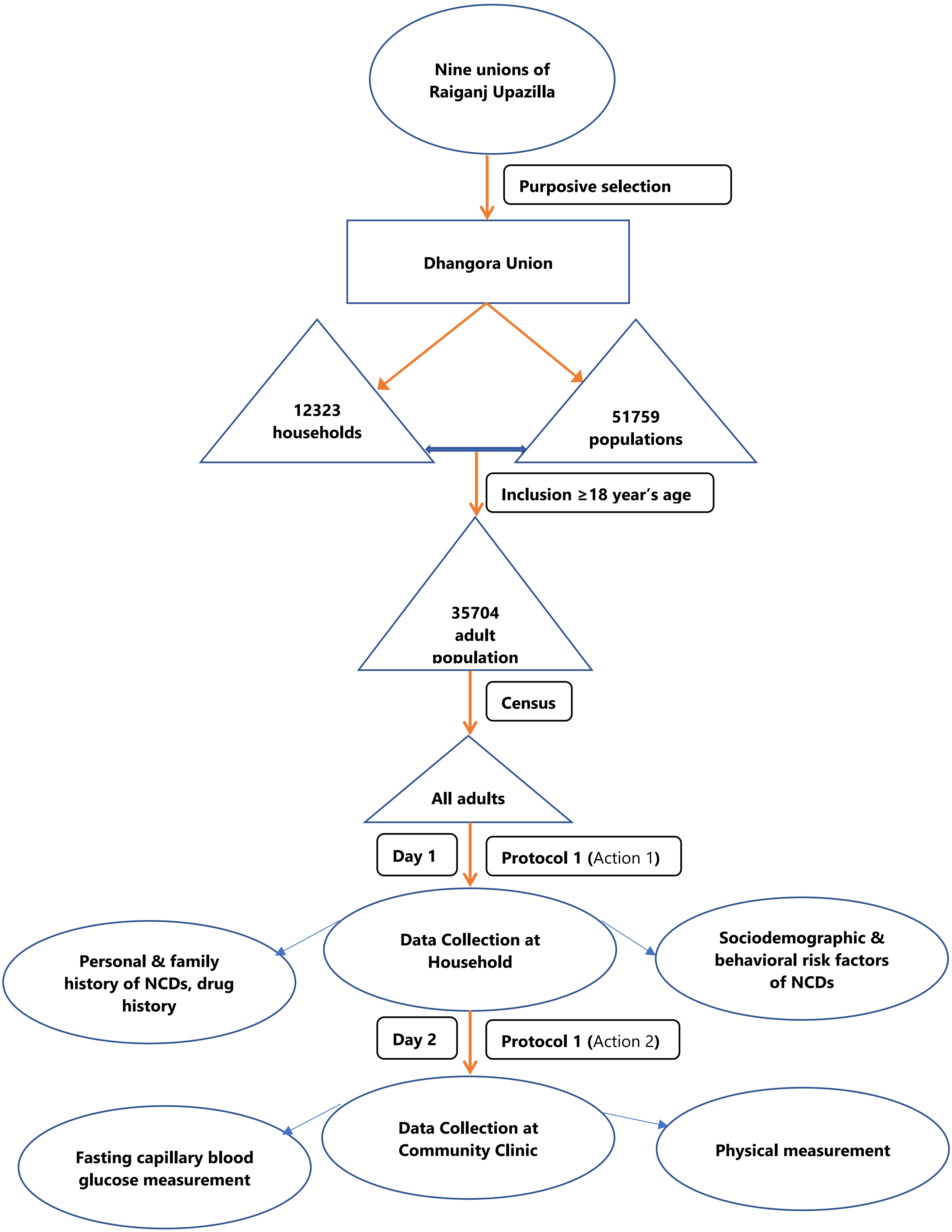

Flow Chart of study methods applied to collect data from selected rural population of Bangladesh
