# Supplementary material for: Baseline prevalence of high blood pressure and its predictors in a rural adult population of Bangladesh: Outcome from the application of WHO PEN interventions
Source: J Clin Hypertens (Greenwich). 2021 Nov 16;23(12):2042–52. doi: 10.1111/jch.14386 (PMC8696237; doi:10.1111/jch.14386)
Supplement: Supplementary file 2 — Supporting information Supportive document 2: WHO PEN Intervention [file JCH-23-2042-s001.pdf]

World Health Organization (WHO) Package of Essential Noncommunicable Disease (PEN) Interventions

WHO PEN Package

- WHO PEN is the minimum standard for NCDs to strengthen national capacity to integrate and scale up care of heart disease, stroke, cardiovascular risk, diabetes, cancer, asthma and chronic obstructive pulmonary disease in primary health care in low-resource settings.
- It is an important first step for integration of NCD into PHC to reform the health systems

Goal

To close the gap between what is needed and what is currently available to reduce the burden, health-care costs and human suffering due to major NCDs by achieving higher coverage of essential interventions in LMIC

Objectives

- To improve the efficiency of care of major NCDs in primary care
- To improve the quality of care of major NCDs in primary care
- To have a beneficial impact on health of the people

Objective-1

- enhanced implementation of human rights standards;
- provision of cost effective interventions based on need rather than ability to pay;
- targeting limited resources to those who are most likely to benefit due to high risk;
- standardization of diagnostic and investigation procedures and drug prescription;
- formulation of referral criteria for further assessment or hospitalization;
- definition of parameters for planning and budget;
- selection of monitoring and evaluation indicators.

Objective-2

- cost effective case management;
- appropriate referral and follow-up;
- prevention, early detection and cost effective case management
- management of exacerbations and emergencies;
- follow-up of long-term treatment prescribed by the specialist.

Objective-3

- reduction of tobacco consumption in NCD patients;
- reduction of the average delay in the diagnosis of NCD by the health services;
- reduction of the risk of heart attacks, strokes, amputations and kidney failure;
- reduction of case fatality of major NCDs;
- prevention of acute events and complications;
- prolongation of the duration of stable clinical periods for CVDs, diabetes, asthma and COPD patients.

Ultimate Goal

TO STRENGTHEN AND ORIENT HEALTH SYSTEMS TO ADDRESS THE PREVENTION AND CONTROL OF NONCOMMUNICABLE DISEASES AND THE UNDERLYING SOCIAL DETERMINANTS THROUGH PEOPLE-CENTRED PRIMARY HEALTH CARE AND UNIVERSAL HEALTH COVERAGE

OBJECTIVE

4

Implementation of WHO PEN is a key component of the objective 4 of the Global Action Plan-

"To strengthen and orient health systems to address the prevention and control of NCDs and the underlying social determinants through people-centred primary health care and universal health coverage"

Ultimate Goal

* A 25% relative reduction in risk of premature mortality from cardiovascular disease, cancer, diabetes or chronic respiratory disease

* Prevention of heart attacks and strokes by providing treatment (including glycemic control) and counselling at least to 50% of eligible people (those with a 10 year cardiovascular risk equal to or above 30%) and reducing their cardiovascular risk.

- Effective implementation of WHO PEN, combined with other very cost effective population-wide interventions, will help to attain the global voluntary targets related to reduction of premature mortality and prevention of heart attack and strokes*.
- Equitable financing of interventions in WHO PEN can be a first step for addressing prevention and control of noncommunicable diseases within the universal health coverage agenda

WHO PEN Protocols for Primary Care

Protocols	Description
Who Pen Protocol 1	Prevention of Heart Attacks, Strokes and Kidney Disease through Integrated Management of Diabetes and Hypertension
WHO PEN Protocol 2	Health Education and Counseling on Healthy Behaviours
WHO PEN Protocol 3	3.1 Management of Asthma<br>3.2 Management of Management of Chronic Obstructive Pulmonary Disease
WHO PEN Protocol 4	4.1 Assessment and referral of women with suspected breast cancer at primary health care<br>4.2 Assessment and referral of women with suspected cervical cancer at primary health care
Essential technologies and tools for WHO PEN	
Core list of medicines for WHO PEN	

WHO PEN Protocol 1

Prevention of Heart Attacks, Strokes and Kidney Disease through Integrated Management of Diabetes and Hypertension

When could this Protocol be used?

- The protocol is for assessment and management of cardiovascular risk using hypertension, diabetes mellitus (DM) and tobacco use as entry points

- It could be used for routine management of hypertension and DM and for screening, targeting the following categories of people:
  - age > 40 years
  - smokers
  - waist circumference (≥ 90 cm in women ≥100 cm in men)
  - known hypertension
  - known DM
  - history of premature CVD in first degree relatives
  - history of DM or kidney disease in first degree relatives

Step-wise Actions of WHO PEN Protocol 1

Action 1 (Ask about)

First Visit		
	○ Diagnosed heart disease, stroke, TIA, DM, kidney disease<br>○ Angina, breathlessness on exertion and lying flat, numbness or weakness of limbs, loss of weight, increased thirst, polyuria, puffiness of face, swelling of feet, passing blood in urine etc<br>○ Medicines that the patient is taking<br>○ Current tobacco use (yes/no) (answer yes if tobacco use during the last 12 months)	○ Alcohol consumption (yes/no) (if `Yes`, frequency and amount)<br>○ Occupation (sedentary or active)<br>○ Engaged in more than 30 minutes of physical activity at least 5 days a week (yes/no)<br>○ Family history of premature heart disease or stroke in first degree relatives

Action 2 (Assess: physical exam and blood and urine tests)

FIRST VISIT	○ Waist circumference<br>○ Measure blood pressure, look for pitting odema<br>○ Palpate apex beat for haeving and displacement<br>○ Auscultate heart (rhythm and murmurs)<br>○ Auscultate lungs (bilateral basal crepitations)<br>○ Examine abdomen (tender liver)<br>○ In DM patients examine feet; sensations, pulses, and ulcers	○ Urine ketones (in newly diagnosed DM) and protein<br>○ Total cholesterol<br>○ Fasting or random blood sugar (diabetes= fasting blood sugar≥7 mmol/l (126 mg/dl)) or random blood sugar ≥11.i mmol/l (200 mg/dl))<br><br>(Point of care devices can be used for testing blood sugar if laboratory facilities are not available)

Action 3 (Estimate cardiovascular risk in those not referred)

FIRST VISIT		
	- Use the WHO/ISH risk charts relevant to the WHO subregion (Annex and CD) - Use age, gender, smoking status, systolic blood pressure, DM (and plasma cholesterol if available) - If age 50-59 years select age group box 50, if 60-69 years select age group box 60 etc., for people age < 40 years select age group box 40 - If cholesterol assay cannot be done use the mean cholesterol level of the population or a value of 5.2 mmol/l to calculate the cardiovascular risk)	- If the person is already on treatment, use pretreatment levels of risk factors (if information is available to assess and record the pretreatment risk. Also assess the current risk using current levels of risk factors) - Risk charts underestimate the risk in those with family history of premature vascular disease, obesity, raised triglyceride levels

Action 4 (Referral criteria for all visits)

FIRST VISIT		
FIRST VISIT	- BP >200/>120 mm Hg (urgent referral)<br>- BP ≥140 or ≥ 90 mmHg in people < 40 years (to exclude secondary hypertension)<br>- Known heart disease, stroke, transient ischemic attack, DM, kidney disease (for assessment, if this has not been done)<br>- New chest pain or change in severity of angina or symptoms of transient ischemic attack or stroke<br>- Target organ damage (e.g. angina, claudication, haeving apex, cardiac failure)<br>- Cardiac murmurs<br>- Raised BP ≥140/90 (in DM above 130/ 80mmHg) while on treatment with 2 or 3 agents	- Any proteinuria<br>- Newly diagnosed DM with urine ketones 2+ or in lean persons of <30 years<br>- Total cholesterol >8mmol/l<br>- DM with poor control despite maximal metformin with or without sulphonylurea<br>- DM with severe infection and/or foot ulcers<br>- DM with recent deterioration of vision or no eye exam in 2 years<br>- High cardiovascular risk<br><br>**If referral criteria are not present go to Action 5**

Action 5 (Counsel all and treat as shown below)

FIRST VISIT	Risk < 20%	○ Counsel on diet, physical activity, smoking cessation and avoiding harmful use of alcohol<br>○ If risk < 10% follow up in 12 months<br>○ If risk 10 - < 20% follow up every 3 months until targets are met, then 6-9 months thereafter
	Risk 20% - 30%	○ Counsel on diet, physical activity, smoking cessation and avoiding harmful use of alcohol<br>○ Persistent BP ≥ 140/90 mm Hg consider drugs (see below ** Antihypertensive medications)<br>○ Follow-up every 3-6 months

Action 5 (Counsel all and treat as shown below)

FIRST VISIT	Risk > 30%	- Counsel on diet, physical activity, smoking cessation and avoiding harmful use of alcohol<br>- Persistent BP ≥ 130/80 consider drugs (see below ** Antihypertensive medications)<br>- Give a statin<br>- Follow-up every 3 months, if there is no reduction in cardiovascular risk after six months of follow up refer to next level
	Important practice points	- Consider drug treatment for following categories<br>- All patients with established DM and cardiovascular disease (coronary heart disease, myocardial infarction, transient ischaemic attacks, cerebrovascular disease or peripheral vascular disease), renal disease. If stable, should continue the treatment already prescribed and be considered as with risk >30%<br>- ** Antihypertensive medications<br>- If under 55 years low dose of a thiazide diuretic and/ or angiotensin converting enzyme inhibitor<br>- If over 55 years calcium channel blocker and/or low dose of a thiazide diuretic<br>- If intolerant to angiotensin converting enzyme inhibitor or for women in child bearing age consider a beta blocker<br>- Thiazide diuretics and/or long-acting calcium channel blockers are more appropriate as initial treatment for certain ethnic groups. Medications for compelling indications should be prescribed, regardless of race/ ethnicity<br>- Test serum creatinine and potassium before prescribing an angiotensin converting enzyme inhibitor\

Additional actions for individuals with DM

- Give an antihypertensive for those with BP ≥130/80 mmHg
- Give a statin to all with type 2 DM aged ≥ 40 years
- Give Metformin for type 2 DM if not controlled by diet only (FBS>7mmol/l), and if there is no renal insufficiency, liver disease or hypoxia.
- Titrate metformin to target glucose value
- Give a sulfonylurea to patients who have contraindications to metformin or if metformin does not improve glycaemic control.
- Give advise on foot hygiene, nail cutting, treatment of calluses, appropriate footwear and assess feet at risk of ulcers using simple methods (inspection, pin-prick sensation)
- Angiotensin converting enzyme inhibitors and/or low-dose thiazides are recommended as first-line treatment of hypertension. Beta blockers are not recommended for initial management but can be used if thiazides or angiotensin converting enzyme inhibitors are contraindicated.
- Follow up every 3 months

Application of WHO PEN Protocol 1 in the Current Study

- **Action 1**: Ask about

  Data collection at the Household level & Referral to Community clinics (CCs)
- **Action 2**: Assess through physical examinations and biochemical tests

  at the CC + Upazilla Health Complexes (UHCs)

- At CC: screening by physical measurements & referral after early detection
- At UHC: physical examinations and confirmation by lab reports

- **Action 3**: CVD risk assessment

- At UHC (By Nurses/Sab-assistant Community Medical Officer (SACMO) at the NCD corner)

- **Action 4**: Refer the cases according to mentioned criteria
- **Action 5**: Counseling and Treat according to CVD risk if not referred

Source of information and more details are available at

Implementation tools: package of essential noncommunicable (PEN) disease interventions for primary health care in low-resource settings

Link: https://mca.essensys.ro/publications/i/item/implementation-tools-package-of-essential-noncommunicable-(-pen)-disease-interventions-for-primary-health-care-in-low-resource-settings

THANK YOU
