# Supplementary material for: Baseline prevalence of high blood pressure and its predictors in a rural adult population of Bangladesh: Outcome from the application of WHO PEN interventions
Source: J Clin Hypertens (Greenwich). 2021 Nov 16;23(12):2042–52. doi: 10.1111/jch.14386 (PMC8696237; doi:10.1111/jch.14386)
Supplement: Supplementary file 3 — Supporting information Supportive document 3: Implementation of WHO PEN intervention phases [file JCH-23-2042-s006.pdf]

Definition of risk factors

Current tobacco user

Those who consumed tobacco in any form in the past 30 days were considered as 'current' user and rest were 'past' user [19].

Current alcohol user

Those who consumed alcohol in the past 30 days were considered as 'current' alcohol user [19].

Inadequate fruits and/ vegetables intake

Less than five servings of fruits and/vegetable a day was considered as inadequate [19].

Added salt intake

The added salt intake was defined as taking dietary salt during eating a meal [23]

Low physical activity

As per the STEPS protocol, we converted all work-related physical activities in metabolic equivalent of task in minutes per day (MET-minute):

1 minute in sedentary position = 1 MET-minute

1 minute in a moderate physical activity = 4 MET-minutes

1 minute in a vigorous physical activity = 8 MET-minutes

All MET-minutes were then added together to get the cumulative MET-minutes. Based on the cumulative MET-minutes, participants were categorized as less, moderately, and highly active [19]:

≤ 600 MET-minutes per week = less active

≈ 600–3000 MET-minutes per week = moderately active

≥ 3000 MET-minutes per week = highly active

Diabetes

Fasting capillary blood glucose ≥ 7.0 mmol/L [19] and/or treatment for raised blood glucose.

Obesity

BMI was used to assess obesity according to the international guidelines [21]. Central obesity was defined according to the cut-off value specified by the International Diabetes Federation (for men, waist-to-hip ratio > 0.90; for women, waist-hip ratio > 0.85) [22].

References

19. Non-Communicable Disease Risk Factor Survey Bangladesh 2010. https://www.who.int/ncds/surveillance/steps/2010_STEPS_Report_Bangladesh.pdf http://www.who.int/chp/steps/bangladesh/en/ (accessed 3 Nov 2019).
21. World Health Organization. Body mass index - BMI. World Health Organization; 2019. http://www.euro.who.int/en/health-topics/disease-prevention/nutrition/a-healthy-lifestyle/body-mass-index-bmi (Accessed, April 4, 2019)
22. World Health Organization. Waist circumference and waist-hip ratio: report of a WHO expert consultation, Geneva, 8-11 December 2008. Geneva: World Health Organization; 2011.
23. Faruque M, Barua L, Banik PC, et al. Prevalence of non-communicable disease risk factors among nurses and para-health professionals working at primary healthcare level of Bangladesh: a cross-sectional study. BMJ Open 2021;11:e043298. doi:10.1136/bmjopen-2020-043298
