# Supplementary material for: Baseline prevalence of high blood pressure and its predictors in a rural adult population of Bangladesh: Outcome from the application of WHO PEN interventions
Source: J Clin Hypertens (Greenwich). 2021 Nov 16;23(12):2042–52. doi: 10.1111/jch.14386 (PMC8696237; doi:10.1111/jch.14386)
Supplement: Supplementary file 4 — Supporting information Supportive document 4: Ethical approval letter [file JCH-23-2042-s005.pdf]

CIPRB

Memo: CIPRB/ERC/2019/003 Date: 23 January, 2019

Ethical Review Committee

Prof. Saidur Rahman Mashreky
Director
Centre for Injury Prevention and
Research Bangladesh (CIPRB)
New DOHS, Mohakhali, Dhaka

Subject: Ethical Clearance

With reference to your application on the above subject, this is to inform you that your research proposal titled "**Designing a model for community-based intervention in reducing hypertension, diabetes and their risk factors in a rural community of Bangladesh**" has been reviewed and approved by the Ethical Review Committee of Centre for Injury Prevention and Research Bangladesh (CIPRB).

You are requested to please note the following ethical guidelines as mentioned at page 2 (overleaf of this memo).

Professor Mahfuzar Rahman
Chairman
Ethical Review Committee

Centre for Injury Prevention and Research, Bangladesh (CIPRB)
House # B-162, Road # 23, New DOHS, Mohakhali, Dhaka-1206

Tel : +88-02-58814988 e-mail : info@ciprb.org
Fax : +88-02-58814964 Web : www. ciprb.org

The ethical Guidelines to be followed by the principal and con investigators

- **The rights and welfare of individual volunteers are adequately protected**
- **The methods to secure informed consent are fully appropriate and adequately safeguard the right of the subjects (in the case of minors, consents are obtain from parents or guardians)**
- **The investigator(s) assume the responsibility of notifying the Ethical Review Committee if there is any change in the methodology of the protocol involving a risk to the individual volunteers**
- **To immediately report Ethical Review Committee if any evidence of unexpected or adverse reaction is noted in the subject under study.**
- **This proposal is subject to P.I's reading and accepting the CIPRB ethical Principles and guidelines currently on operation.**
