# Supplementary material for: Baseline prevalence of high blood pressure and its predictors in a rural adult population of Bangladesh: Outcome from the application of WHO PEN interventions
Source: J Clin Hypertens (Greenwich). 2021 Nov 16;23(12):2042–52. doi: 10.1111/jch.14386 (PMC8696237; doi:10.1111/jch.14386)
Supplement: Supplementary file 5 — Supporting information Supportive Figure S1: Map of Bangladesh & the study site [file JCH-23-2042-s004.pdf]

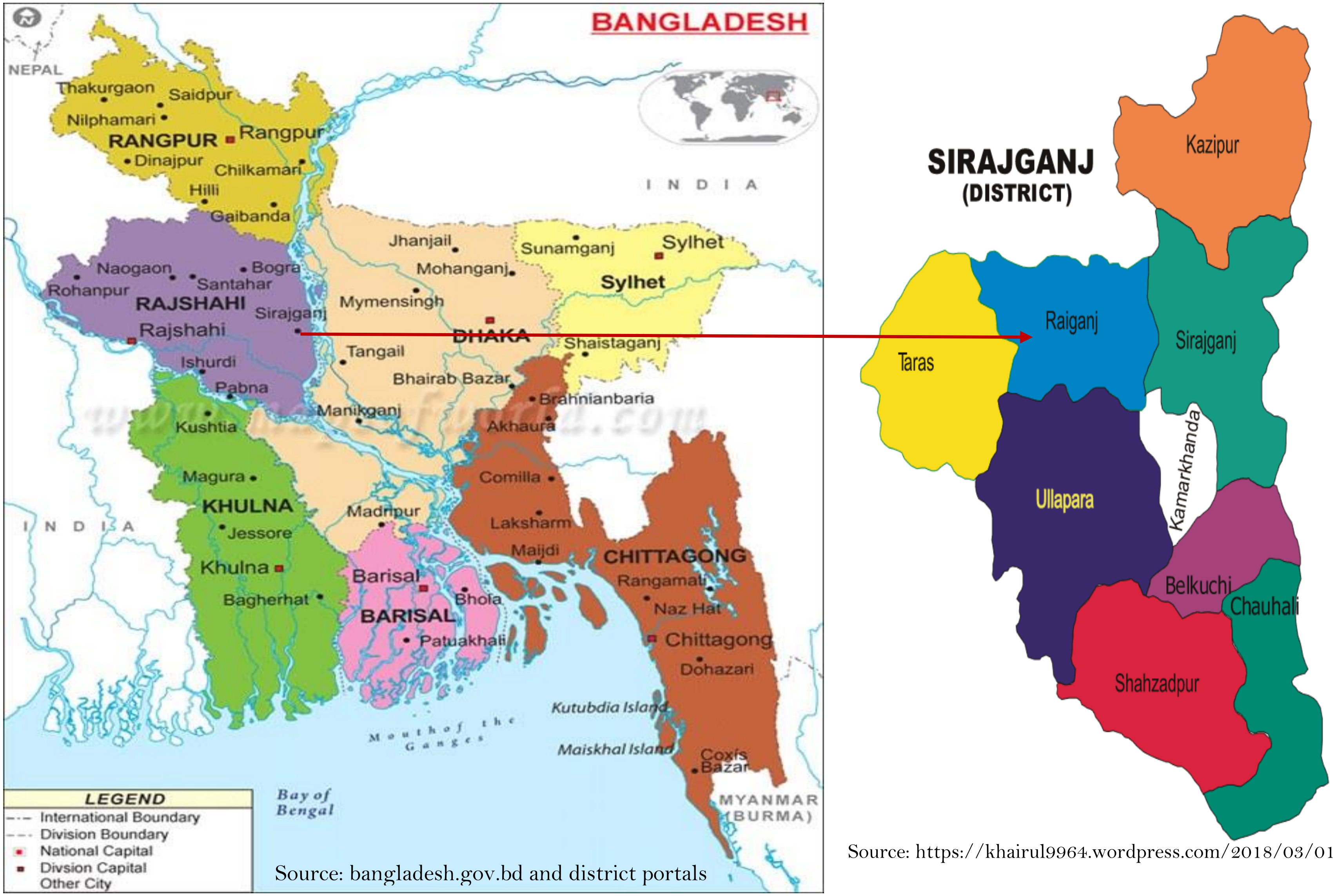

Source: bangladesh.gov.bd and district portals

Source: https://khairul9964.wordpress.com/2018/03/01/sirajganj/

Figure S1: Map of Bangladesh showing the site of study
