# Supplementary material for: Baseline prevalence of high blood pressure and its predictors in a rural adult population of Bangladesh: Outcome from the application of WHO PEN interventions
Source: J Clin Hypertens (Greenwich). 2021 Nov 16;23(12):2042–52. doi: 10.1111/jch.14386 (PMC8696237; doi:10.1111/jch.14386)
Supplement: Supplementary file 6 — Supporting information Supportive Table S1: Univariate analysis (χ2‐test) between the blood pressure categories (normal/pre‐hypertension/hypertension) and the risk factors among Bangladeshi rural adults, n = 11145 [file JCH-23-2042-s002.pdf]

Table S1: Univariate analysis (χ^2-test) showing the association of blood pressure categories (normal/pre-hypertension / hypertension) with the risk factors among Bangladeshi rural adults, n = 11145

Risk factors	Categories of risk factors	High blood pressure (BP) categories (%)			χ^2-value	*P*-value
		Normal*	Pre-HTN†	HTN‡		
Age	≥ 40 years	38.6	24.9	36.5	1108.1	< 0.001
	< 40 years	64.4	25.9	9.7		
Gender	Women	49.0	23.6	27.4	45.7	< 0.001
	Men	48.5	28.7	22.8		
Education	Literate	51.9	25.4	2.7	114.8	< 0.001
	Illiterate	43.3	25.3	31.5		
Family history of HTN	Present	44.8	26.8	28.4	26.8	< 0.001
	Absent	50.3	24.8	24.9		
Tobacco use	Yes	47.4	24.4	28.2	18.2	<0.001
	No	49.6	25.8	24.5		
Alcohol intake (once in life)	Yes	57.1	30.0	12.9	6.2	0.05
	No	48.8	25.3	25.9		
Inadequate fruit/vegetable intake	Yes	49.3	24.5	26.2	8.4	0.02
	No	47.7	27.1	25.2		
Fast food intake	Yes	52.7	28.2	19.1	121.8	< 0.001
	No	47.1	24.0	28.9		
Sweetened beverage intake	Yes	52.3	27.0	20.7	90.5	< 0.001
	No	46.8	24.3	28.9		
Added salt intake	Yes	51.8	26.0	22.2	137.9	< 0.001
	No	43.8	24.1	32.1		
Physical activity	Inactive	40.7	26.1	33.3	86.3	< 0.001
	Active	50.6	25.2	24.2		
Central obesity	Present	44.5	25.9	29.6	322.1	< 0.001
	Absent	62.1	23.5	14.3		
Generalized obesity	Present	23.0	30.9	46.0	124.1	< 0.001
	Absent	49.8	25.1	25.1		
Diabetes	Present	22.5	23.9	53.5	492.0	< 0.001
	Absent	51.5	25.5	23.1		

* Systolic blood pressure < 120 mmHg and diastolic blood pressure < 80 mmHg
†Systolic blood pressure 120 – 139 mmHg or diastolic blood pressure 80 -89 mmHg
‡Systolic blood pressure ≥ 140 mmHg and/ diastolic blood pressure ≥ 90 mmHg
